# Sonoporation of the Round Window Membrane on a Sheep Model: A Safety Study

**DOI:** 10.3390/pharmaceutics15020442

**Published:** 2023-01-29

**Authors:** Sandrine Kerneis, Jean-Michel Escoffre, John J. Galvin, Ayache Bouakaz, Antoine Presset, Corentin Alix, Edward Oujagir, Antoine Lefèvre, Patrick Emond, Hélène Blasco, David Bakhos

**Affiliations:** 1 ENT and Cervico-Facial Surgery Department, University Hospital Center of Tours, 2 Boulevard Tonnellé, 37044 Tours, France; 2 UMR 1253, iBrain, Inserm, Université de Tours, 10 Boulevard Tonnellé, 37044 Tours, France; 3 Faculty of Medicine, Université de Tours, 10 Boulevard Tonnellé, 37000 Tours, France; 4 House Institute Foundation, 2100 W 3rd Street, Suite 111, Los Angeles, CA 90057, USA; 5 Department of Biochemistry and Molecular Biology, University Hospital Center of Tours, 2 Boulevard Tonnellé, 37044 Tours, France

**Keywords:** sonoporation, round window membrane, metabolomics, perilymph, inner ear

## Abstract

Sonoporation using microbubble-assisted ultrasound increases the permeability of a biological barrier to therapeutic molecules. Application of this method to the round window membrane could improve the delivery of therapeutics to the inner ear. The aim of this study was to assess the safety of sonoporation of the round window membrane in a sheep model. To achieve this objective, we assessed auditory function and cochlear heating, and analysed the metabolomics profiles of perilymph collected after sonoporation, comparing them with those of the control ear in the same animal. Six normal-hearing ewes were studied, with one sonoporation ear and one control ear for each. A mastoidectomy was performed on both ears. On the sonoporation side, Vevo MicroMarker^®^ microbubbles (MBs; VisualSonics—Fujifilm, Amsterdam, The Netherlands) at a concentration of 2 × 10^8^ MB/mL were locally injected into the middle ear and exposed to 1.1 MHz sinusoidal ultrasonic waves at 0.3 MPa negative peak pressure with 40% duty cycle and 100 μs interpulse period for 1 min; this was repeated three times with 1 min between applications. The sonoporation protocol did not induce any hearing impairment or toxic overheating compared with the control condition. The metabolomic analysis did not reveal any significant metabolic difference between perilymph samples from the sonoporation and control ears. The results suggest that sonoporation of the round window membrane does not cause damage to the inner ear in a sheep model.

## 1. Introduction

Inner ear disorders are responsible for hearing loss, tinnitus, or vertigo. These pathologies are common and can severely impact patients’ health. Hearing loss is the second leading cause of disability worldwide after depression [1] and is the leading modifiable risk factor for dementia [2]. The delivery of therapeutic drugs to the inner ear to treat these pathologies is challenging. The inner ear is a fragile organ, encircled by a dense bone, which offers limited access. Systemic delivery of therapeutic drugs is not an optimal administration route [3]. Indeed, its effectiveness is limited by the presence of the blood-cochlear barrier, the high density of the peri-cochlear bone, and poor peri-cochlear vascularization. Moreover, this administration route of therapeutics induces side effects, including, for example, systemic administration of corticosteroids for diabetic patients.

Intratympanic injection allows for better delivery of therapeutic drugs to the inner ear [4,5] and results in fewer side effects compared with systemic delivery. Molecules injected in the middle ear diffuse to the inner ear, mainly through the round window membrane (RWM), and secondarily through the annular ligament, around the stapes [6,7]. The intratympanic approach is commonly used to treat several diseases. For example, intratympanic injections of gentamicin [8] and corticosteroids [9,10] are used to treat Meniere’s disease. Intratympanic injection of corticosteroids is also used to treat sudden sensorineural hearing loss [11,12], acoustic shock, tinnitus, and auto-immune deafness [13,14]. However, the effectiveness of this approach is variable because the intratympanic delivery of drugs is not well controlled. More efficient and targeted delivery methods are required to increase the local concentration of antibiotics and corticosteroids in the endo-cochlear fluids while reducing off-target effects.

Alternative methods have been designed and validated to improve the delivery of therapeutic drugs through the RWM to the hair cells of the inner ear and the spiral ganglion, such as drug-loaded nanoparticles [15,16,17], liposomal gels [18], and extracellular vesicles [19]. Drug-eluting coating on cochlear implant electrodes [20], as well as perfusion systems with micro-pumps or reservoirs connected directly into cochlear implants, are also under investigation [21,22]. Other therapeutic strategies rely on the transduction of therapeutic genes into target tissues using viral vectors [23]. To date, these approaches are being validated in preclinical models or even undergoing clinical validation but are not yet recommended to treat patients.

Recently, sonoporation-mediated drug delivery has shown great promise in improving the efficacy of therapeutic drugs (e.g., chemotherapeutic drugs, therapeutic antibodies, antibiotics, nucleic acids, etc.) by enhancing their local deposition and reducing off-target effects [24]. Sonoporation uses a combination of high-frequency ultrasound (1–10 MHz) and ultrasound contrast agents (i.e., gas microbubbles (MBs)). The acoustically mediated volumetric oscillations of the MBs induce several local acoustic events near the biological barriers (e.g., cell plasma membrane, endothelial barriers, etc.), thereby enhancing their permeability for therapeutic molecules in a transient, localised, and non-invasive manner. As such, sonoporation improves intra-tissue bioavailability, thereby increasing therapeutic efficacy [25].

Recently, the permeabilization of RWM to fluorescent markers (Biotin-FITC, Texas-red-labelled gentamicin) or therapeutic molecules (plasmid DNA, dexamethasone, and insulin growth factor 1) using sonoporation has been successfully reported in vitro and in guinea pig models [26,27,28,29,30,31,32]. These studies described acoustic protection when applying dexamethasone [28,31] and a better uptake by vestibular hair cells when applying gentamicin [29]. The efficiency of acoustically mediated drug delivery depends on the application route of ultrasound (e.g., transcanal or transcranial) and ultrasound parameters, including the central frequency, the duty cycle, the acoustic intensities, and the exposure time [29,30]. No auditory damage or irreversible histological changes to the RWM related to sonoporation have been reported [26,28,29,30,31,32]. In addition, no acoustically induced thermal toxicity (threshold of 1.5 °C [33]) has been observed when the ultrasound parameters were perfectly controlled [29,30]. However, metabolomics profiles of perilymph were not used in these previous studies to confirm the absence of ototoxicity. Furthermore, while these studies demonstrated that sonoporation could enhance the passage of therapeutic molecules to the inner ear, guinea pig models were used. The guinea pig has substantial limitations as a model of the human ear and RWM, as the RWM thickness is 10–15 μm [34], compared with 70 μm in humans [35]. Sheep may be a more relevant large animal model for studies of the human auditory system. The anatomical structures of the sheep’s inner, middle, and outer ears are comparable with those of humans [36,37,38,39]. Similarities between the sheep ear and the human ear have also been found in terms of the histological composition of temporal bone structures [40], the anatomy and histology of the RWM [41], the auditory spectrum [41], and the recording of auditory brainstem responses (ABRs) [42].

The objective of the present study was to assess the safety of sonoporation of the inner ear in a sheep model. To achieve this objective, we assessed auditory function and cochlear heating and analysed metabolomics profiles of perilymph and plasma with and without sonoporation of RWM.

## 2. Material and Methods

### 2.1. Animals and Housing

All six sheep were of the Ile de France breed (PIXANIM platform, INRAE, Nouzilly, Centre Val-de-Loire, France). The mean age was 28 ± 4 months, and the mean weight was 60 ± 5 kg. Only female sheep (ewes) were used because their temporal bone is thinner than that of rams, which facilitates drilling of the temporal bone. The auditory cortex in sheep is mature by 12 months. For the reason that we expected large inter-individual variability in the perilymph used for metabolomic analysis [43], each sheep served as its own control, with one sonoporation ear and one control ear; sonoporation and control ears were randomised across sheep. The number of sheep (n = 6) allowed for sufficient statistical power for the metabolomic study. Indeed, this number has been shown to be sufficient to demonstrate metabolomic changes in the perilymph following acoustic trauma in sheep [44].

Twenty-four hours before the experiment, the sheep were isolated and fasted. The animal study was reviewed and approved by the Animal Care and Regional Committee for Ethics in Animal Experiments, Centre Val-de-Loire (APAFiS code #33161-2021091518241644) in accordance with European Directive 2010/63/EU for animal experiments.

### 2.2. Sonoporation Protocol

**Figure 1:**
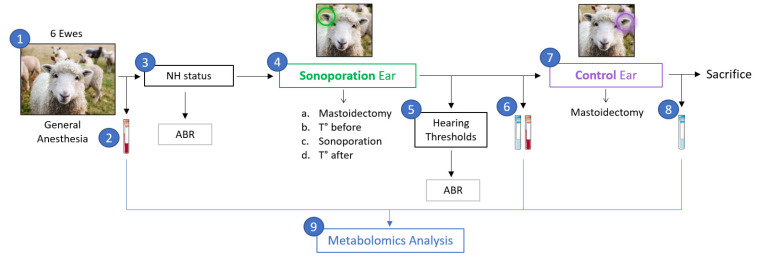
Study protocol. (1) 6 sheep under general anesthesia. (2) Plasma sample after anesthesia (before sonoporation). (3) Verification of NH status using ABR. (4) Sonoporation ear: a. Mastoidectomy, b. Measure of promontory temperature before sonoporation, c. Sonoporation of ultrasound activated MBs injected into the middle ear, and d. Verification of promontory temperature after sonoporation. (5) Verification of hearing thresholds. (6) Perilymph and plasma sampling. (7) Control ear: mastoidectomy. (8) Perilymph sampling before sacrifice of the animal. (9) Metabolomic analysis of the samples.

The general procedure is presented in Figure 1. In the protocol, the six sheep were put under general anesthesia, and plasma samples were collected. Furthermore, verification of normal-hearing (NH) status was confirmed using ABRs. For the sonoporation ear, a mastoidectomy was performed, and the promontory temperature was measured before sonoporation. Furthermore, sonoporation of ultrasound-activated MBs was performed in the middle ear. Promontory temperature and hearing thresholds were re-measured after sonoporation. Finally, perilymph and plasma were collected after sonoporation. For the control ear, a mastoidectomy was performed and perilymph was collected. At the end of the experiment, all the sheep were sacrificed. The perilymph and plasma samples were then subjected to metabolomics analysis.

#### 2.2.1. Surgery

**Figure 2:**
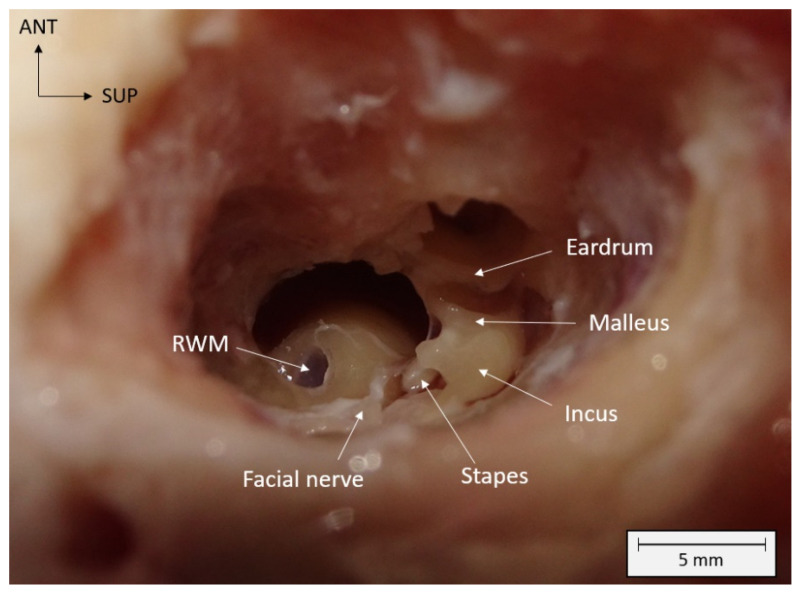
Visualization of the round window membrane (RWM) and anatomical structures of the middle ear after mastoidectomy on a left ewe ear. Spatial orientation is shown with anterior (ANT) and superior (SUP) localizations. mm: millimeters.

Under general anesthesia (inhalation of isoflurane at 3% and intraveinous ketamine injection at 10 mg/kg), the sheep were intubated and placed in the lateral decubitus position. A surgery similar to a human mastoidectomy was performed. The mastoid of the ewe is unventilated and does not contain mastoid cells. The mastoid was milled following the external auditory canal, taking care not to damage the lateral sinus posteriorly or the temporal meninge superiorly. Once the middle ear was reached, the ossicular chain was respected, and the milling continued posteroinferiorly to open the hypotympanic bulla and to reveal the RWM. Direct visualisation was possible by sacrificing the facial nerve and the pyramid (Figure 2).

#### 2.2.2. Ultrasound Treatment

As previously described [45], Vevo MicroMarker^®^ MBs (VisualSonics—Fujifilm, Amsterdam, the Netherlands) are composed of a mixture of nitrogen and perfluorobutane gases encapsulated in a thin and flexible monolayer of phospholipids. MBs were prepared according to the manufacturer’s instructions (with 1 min of hand stirring and 10 min of resting at room temperature) and then diluted 10 times to reach a concentration of 2 × 10^8^ MB/mL. This MB concentration is similar to that exploited in previous sonoporation studies of the inner ear, which used phospholipid MBs (SonoVue^®^, Bracco Group, Milan, Italy) [28,30,32]. A solution of MBs with an average volume of 886 ± 421 μL (range: 420–1680 μL, depending on the size of the mastoidectomy) was injected into the middle ear. No leakage of MBs through the eustachian tube was observed under an optical microscope before or during sonoporation.

**Figure 3:**
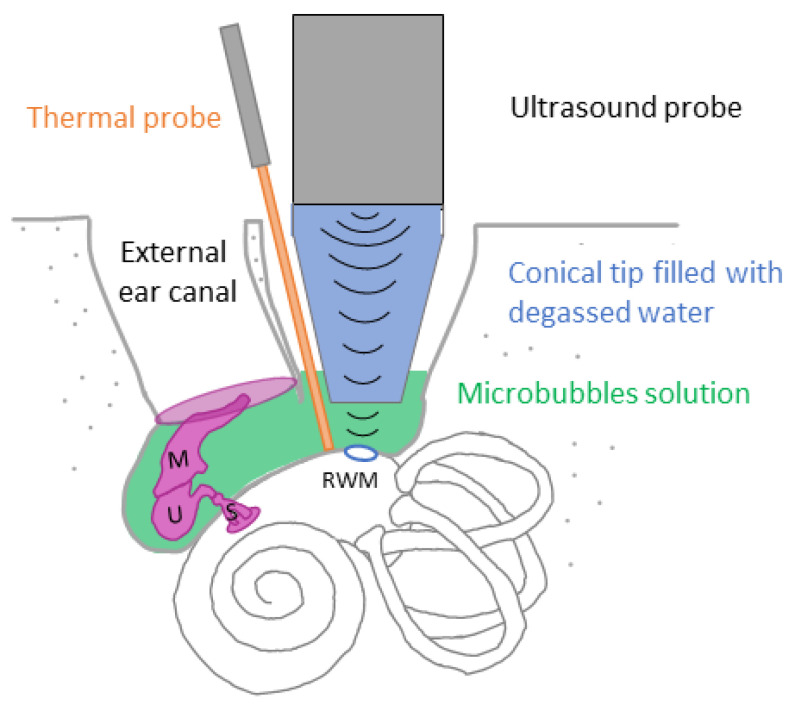
Diagram of the sonoporation of RWM. After surgical mastoidectomy, MBs solution (in green) was injected in the middle ear and ultrasound were applied using an ultrasound probe connected to a degassed water-filled cone. Promontory temperature was measured before and after sonoporation using a thermal probe (in orange). M: Malleus, U: Uncus, S: Stapes, RWM: Round window membrane.

Ultrasound waves were generated using a single-element lab-made transducer with a center frequency of 1.1 MHz, which was introduced into the mastoidectomy cavity. The transducer had a diameter of 20 mm and was naturally focused at 30 mm. The transducer was connected to a degassed, water-filled cone to ensure coupling with the RWM (Figure 3).

The RWM was exposed to 40 cycles of 1.1 MHz sinusoidal ultrasound waves repeated every 100 μs (i.e., 40% duty cycle) at a peak-negative pressure of 300 kPa for 1 min. This ultrasound sequence was repeated three times, with 1 min between each application to prevent tissue heating. No stirring method was used during sonoporation. For the control ear, MBs were not injected, and ultrasound waves were not applied. At the end of the procedure, the animals were euthanised by an intravenous injection of a lethal dose of Doléthal^®^ (15–20 mL; Laboratoire Vetoquinol, Lure, France).

### 2.3. Assessment of Auditory Function

Auditory thresholds were assessed using ABRs (NavPRO ONE Bio-logic^®^, Otometrics, Natus Medical Inc., Middleton, WI, USA), with stimulation via a B71W bone transducer and responses recorded via subcutaneous needle electrodes. The stimuli were clicks at a modulation frequency of 30.7 Hz. Auditory thresholds were measured from 50 dB (transducer’s limit) to 20 dB, with 40 dB of contralateral air-conduction masking. Response waves I–IV were obtained. The threshold of disappearance of wave IV was measured, and wave IV latencies were calculated. Note that no wave V is found in sheep. ABRs were recorded bilaterally at the beginning of the procedure and after sonoporation in the sonoporation ear.

### 2.4. Temperature Measurement

A Dotsmann^®^ P655-LOG digital thermometer with a Pt100 thermocouple was used to measure cochlear heating. The device was calibrated according to the manufacturer’s instructions and has an accuracy of ±0.05 °C from −200 °C to +200 °C. The thermocouple was placed in contact with the promontory to measure cochlear heating. The thermal measurement was performed twice on the sonoporation ear: (1) after mastoidectomy but before sonoporation and (2) immediately after sonoporation and aspiration of the MB solution (i.e., 5–10 s after sonoporation ended). With this method, the ultrasound beam did not interact with the thermocouple, inducing no artifact upon the temperature measurement. The light of the surgical microscope was turned off at the end of the mastoidectomy and after MB aspiration to limit artificial overheating.

### 2.5. Metabolomic Analysis

#### 2.5.1. Sample Collection

Perilymph sampling was performed for the sonoporation and control ears through the RWM. After incision of the RWM with a 22G needle, the perilymph was collected by capillary action (Microcap 15 μL, length 54 mm, Drummond^®^) to obtain at least a volume of 1 µL. A 5 mL blood sample was collected after induction of anesthesia (before sonoporation) and a second sample was collected immediately after the sonoporation procedure. The samples were collected in heparinised tubes and immediately centrifuged (1509× *g* at 4 °C for 13 min), and the plasma was collected. All samples were collected in 2 mL polypropylene tubes and placed on dry ice before being stored at −80 °C. The procedures were all performed in the morning to avoid a possible bias related to the nychthemeral cycle of the perilymph composition.

#### 2.5.2. Sample Analysis

The samples were prepared as described by Mavel et al. [46]. Briefly, a volume of 400 μL of methanol is added to 50 μL of perilymph or plasma in order to extract metabolites. The samples were stirred for 5 sec and then incubated at −20 °C for 30 min to deproteinise them. After centrifugation (4193× *g* at 4 °C for 25 min), the supernatant (350 μL) was collected and evaporated with a SpeedVac concentrator (Labconco Corporation, Kansas City, MO, USA) at 40 °C. The dry residue was resuspended in 100 μL of methanol/water (75/25) and then 5 μL of the sample was injected into the liquid chromatography column for analysis.

As described by Diémé et al. [47], the analyses were performed on an UPLC Ultimate WPS-3000 system (Dionex, Germering, Germany) coupled to a Q-Exactive mass spectrometer (Thermo Fisher Scientific, Bremen, Germany). Liquid chromatography (LC) was performed at 40 °C using two modalities: HILIC (Hydrophilic Interaction Liquid Chromatography) and C18, using complementary stationary phases. Ionisation was performed in the positive electrospray mode (ESI+) for C18 and HILIC and the negative electrospray mode (ESI-) for C18, thus giving results in three modalities. Two mobile phases were used, and the chromatographic gradients were run at a flow rate of 0.3 mL/min. During acquisition, the instrument was operated at a resolution of 70,000 (*m*/*z* = 200). The successive use of LC and High-Resolution Mass Spectrometry (HRMS) allowed the separation of compounds from each sample according to their retention time in the LC column for each of the three modalities and according to their molecular weight, allowing for the identification of a peak intensity for each compound.

Identification of metabolites from each sample was performed using an MSML^®^ standard compound library (Mass Spectrometry Metabolite Library of Standards: IROA Technologies™, Sea Girt, NJ, USA). To identify the metabolites, several criteria were used: the retention time of the metabolite had to be within 20 s of the reference, and the molecular weight had to be within 10 ppm of the reference. The signal intensity value was calculated using Xcalibur^®^ 2.2 software (ThermoFisher Scientific, San Jose, CA, USA) by integrating the chromatography peaks corresponding to the selected metabolites. 

The stability of the LC and mass spectrometry instruments was assessed by multiple quality control (QC) samples obtained from a 10 mL pool of an equal volume mixture of all samples analysed. QCs were analysed at the start of the assay, then every 10 samples, and at the end of the assay. Coefficients of variation (CVs, defined as standard deviation/mean × 100) were calculated for each metabolite found in the test and control samples. Metabolites with a CV QC > 30% or >the sample CV were considered false positives and excluded from further analysis.

### 2.6. Statistical Analysis

#### 2.6.1. Statistical Analysis of Auditory Function 

Statistical analyses were performed using Prism 9^®^ version 9.4.1 (GraphPad Software ©, Inc., Boston, MA, USA). A two-sided non-parametric Wilcoxon matched pairs signed rank test with a 95% confidence interval was used to compare the wave IV thresholds and latencies of the sonoporation ear before and after sonoporation. Latencies were analysed using a one-way analysis of variance (ANOVA) with three factors: the sonoporation ear before sonoporation, the sonoporation ear after sonoporation, and the control ear. For all tests, a *p*-value < 0.05 indicated a significant difference. A significantly higher threshold or longer latency after sonoporation indicated a deterioration in hearing.

#### 2.6.2. Statistical Analysis of Temperature Measurements

Statistical analyses were performed using Prism 9^®^ version 9.4.1 (GraphPad Software^©^, Inc., Boston, MA, USA). Temperature measurements were compared using a two-tailed non-parametric Wilcoxon matched-pairs signed rank test with a 95% confidence interval. A *p*-value < 0.05 indicated a significant difference.

#### 2.6.3. Statistical Analysis of Metabolomics Samples

Statistical analyses were performed using R^®^ software version 4.2.1. (Bell Laboratories, Inc., Windsor, WI, USA) The data collected in the three modalities were pooled. In cases of redundancy in the identification of metabolites, the modality with the lowest CV QC was chosen for the analysis.

Analyses of the metabolites were conducted using multivariate and univariate analysis. Unsupervised multivariate analysis consisted of a principal component analysis (PCA) to (1) assess the distribution of the samples, (2) group similar samples, and (3) identify outliers. The PCA represents the main directions in which the samples vary. The main dimensions (p1, p2, and p3) explained the maximum variance of the samples. Conventionally, p1 and p2 are used for graphical representations and can be used to summarise a large amount of data into a two-dimensional space.

Supervised multivariate analysis in Partial Least-Squares Discriminant Analysis (PLS-DA) was used to identify a predictive model to differentiate groups by constraining the samples to their own group. The significance of the model established was assessed by two *p*-values: pR2Y and pQ2; the model was significant if these *p*-values were <0.05. The predictivity of the model was evaluated by the value of Q2, which must be >0.5. This supervised analysis determined a VIP (Variable Importance in Projection) for each metabolite, which summarised the importance of each metabolite in the predictivity of the model. Metabolites with a VIP > 1 were retained, and metabolites significantly impacting metabolic pathways were sought using MetaboAnalyst V5.0 (www.metaboanalyst.ca, accessed on 22 January 2023). 

Univariate analysis was performed using paired Student’s *t*-tests for each metabolite, assuming a normal distribution. A False Discovery Rate (FDR) method was used to adjust p-values for multiple comparisons. Metabolites with adjusted *p*-values < 0.05 were considered to be significantly altered between groups. If metabolites were significantly altered, MetaboAnalyst V5.0 was used to search for significantly impacted metabolic pathways.

Each significant pathway found after multivariate or univariate analysis was examined for relevance and consistency with pathophysiological hypotheses.

## 3. Results

### 3.1. Evaluation of Auditory Thresholds

**Figure 4:**
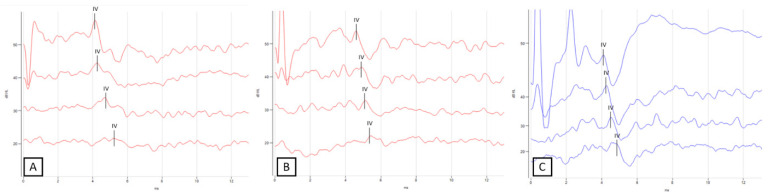
Representative measurement of bone conduction ABR for sonoporation side (right ear) before (**A**) and after sonoporation (**B**) and control side (left ear) (**C**) for sheep #5. Wave IV can be clearly observed and was not modified by sonoporation.

ABR was used to assess the hearing function of all sheep. Figure 4 shows the results of the recordings for sheep #5, as an example.

The mean threshold for wave IV was 23.3 ± 5.16 dB before sonoporation, 25.0 ± 5.47 dB after sonoporation, and 23.3 ± 5.16 dB for the control ear. ABR thresholds are presented in Table 1. For all sheep, thresholds were unchanged, except for sheep #3, where the wave IV threshold increased from 20 dB before sonoporation to 30 dB after sonoporation. A Wilcoxon matched-pairs signed-rank test did not show any significant changes in thresholds after sonoporation compared with before sonoporation (*p* > 0.99; W = −1.00) or to the control ear (*p* > 0.99; W = 1. 00).

Latency measurements at 30 and 20 dB are presented in Table 2. Wilcoxon matched pairs signed rank tests showed no significant differences in latency before or after sonoporation at 30 dB (*p* = 0.34; W = −10.00) or 20 dB (*p* = 0.25; W = −6. 00). A one-way ANOVA of the 30 dB latency data showed no significant difference in latency before sonoporation, after sonoporation, or to the control ear [F(2, 17) = 0.8; *p* = 0.457]. A one-way ANOVA of the 20 dB latency data showed no significant difference in latency before sonoporation, after sonoporation, or to the control ear [F(2, 10) = 0.8; *p* = 0.479].

### 3.2. Temperature Measures

The mean temperature measured at the promontory was 33.8 ± 3.65 °C and 34.0 ± 3.57 °C before and after sonoporation, respectively. A Wilcoxon matched pairs signed rank tests showed no significant increase in temperature after sonoporation (mean difference = 0.21 ± 0.47 °C; *p* = 0.438; W= −9.00). 

### 3.3. Metabolomics Analysis of Perilymph

#### 3.3.1. Perilymph Composition

Metabolomic analysis with LC-HRMS found 201 metabolites, of which 84 were detected in C18 Negative Mode, 63 in C18 Positive Mode, and 107 in HILIC Positive Mode. After the selection of duplicates and the exclusion of metabolites with QC CVs > 30% or >sample CVs, the relative concentrations of 198 metabolites in the 12 samples were retained for statistical analysis. The list of these metabolites is presented in Supplementary Table S1.

#### 3.3.2. Perilymph Comparison by Multivariate Analysis

We conducted the multivariate analysis with and without adjustment for the data from samples S1 (sonoporation side of sheep #1) and C6 (control side of sheep #6). These samples were contaminated by blood, and therefore, some metabolite concentrations were artificially elevated. To avoid bias due to the extreme values found in these samples, we calculated for each of their metabolites the percentage difference between the sample and the average across the other samples of the same group (sonoporation or control). Only the metabolites showing less than a 50% difference relative to the average of the other samples were retained. 

**Figure 5:**
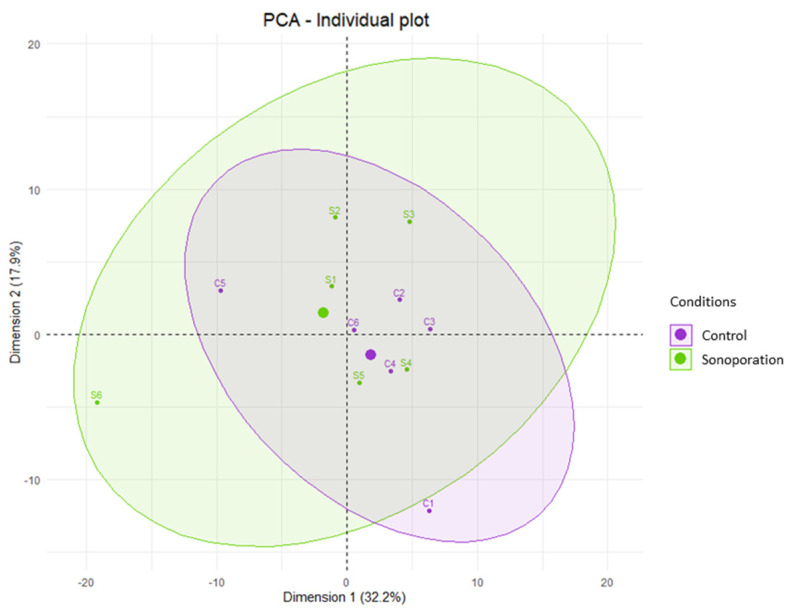
Unsupervised multivariate analysis of perilymph comparing data from sonoporation and control groups. Score-plot of the PCA is shown with adjustment of S1 and C6 data constructed from the metabolites found according to the first two components p1 and p2. The control samples are in purple (C1 to C6) and the sonoporation samples are in green (S1 to S6). Each group is associated with an ellipse regrouping all its samples. The thicker dots correspond to the center of each ellipse. Two samples with the same metabolomic profile appear grouped on this graph (i.e., samples S4 and C4).

The PCA analysis showed that p1 explained 32.2% of the variance and p2 explained 17.9% of the variance. The PCA score plot representing the distribution of the samples according to p1 and p2 is shown in Figure 5. The ellipses are overlapped, and the samples grouped toward the center of the graph show a common metabolomic profile. The PCA according to the other principal components (p2 and p3, p1 and p3) confirmed this profile. 

We conducted the PLS-DA analysis using p1 and p2 with 100 permutations. The PLS-DA metrics revealed a non-significant predictive model with pR2Y = 0.89, pQ2 = 0.91 and Q2 = −0.89. Thus, there were no predictive metabolites to classify the samples into the post-sonoporation or control groups. Even when the data were not adjusted for the outliers from S1 and C6, PLS-DA analysis did not result in a significant predictive model, with pR2Y = 0.35, pQ2 = 0.87, and Q2 = −0.59. The PCA score plot representing the distribution of all samples according to p1 and p2 is presented in Supplementary Figure S1.

#### 3.3.3. Perilymph Comparison by Univariate Analysis

**Figure 6:**
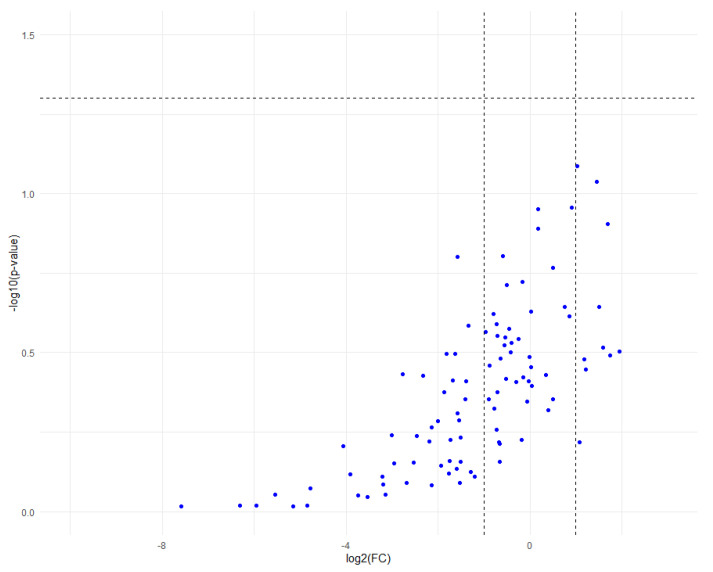
Volcano plot representing metabolite dysregulations between control and sonoporation groups, with thresholds set at log2FC < −1 or >1, and −log10 (*p*-value) > 1.30 (*p* < 0.05). The vertical dotted bars represent the log2FC thresholds at −1 and 1. As all metabolites were under the horizontal dotted line, none of the expression variations were significant. The graph was similar using the FDR adjusted *p*-value.

The univariate analysis was conducted using student *t*-tests on the relative concentrations of the 198 metabolites found in the 12 samples. The relative concentration of each metabolite was compared between the control group and the sonoporation group. Analysis (before or after adjustment for multiple comparisons by the FDR test) did not find any metabolite significantly modified between groups (Figure 6). 

### 3.4. Metabolomics Analysis of Plasma

#### 3.4.1. Plasma Composition

Metabolomic analysis with LC-HRMS found 269 metabolites, of which 99 were detected in C18 Negative Mode, 83 in C18 Positive Mode and 167 in HILIC Positive Mode. After the selection of duplicates and the exclusion of metabolites with QC CVs > 30% or >sample CVs, we retained for analysis the relative concentrations of 267 metabolites in the 12 samples. 

#### 3.4.2. Plasma Comparison before and after Sonoporation by Multivariate Analysis

**Figure 7:**
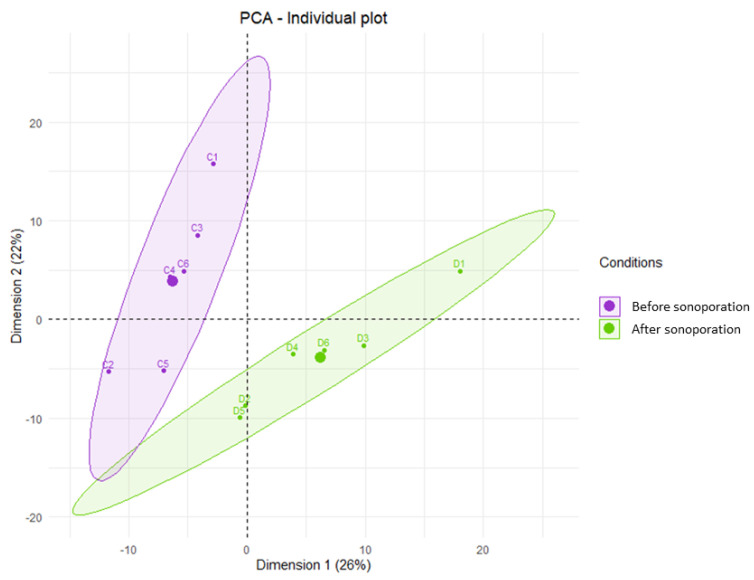
Unsupervised multivariate metabolomic analysis of plasma collected before and after sonoporation. The plot score of the PCA was constructed from the metabolites found according to components p1 and p2. Pre-sonoporation samples are shown in purple (C1 to C6) and post-sonoporation samples are shown in green (D1 to D6). Each group is associated with an ellipse grouping all its samples. The thicker dots correspond to the center of each ellipse.

The PCA analysis showed that p1 explained 26% of the variance and p2 explained 22% of the variance. The score-plot of the PCA (Figure 7) according to the principal components p1 and p2 did not find any outlier samples and separated the plasma samples taken before and after sonoporation into two distinct groups. PCA according to the other principal components (p2 and p3, p1 and p3) confirmed these divergent metabolomic profiles. 

We conducted a PLS-DA analysis using p1 and p2 with 100 permutations. The metrics revealed a significantly predictive model with pR2Y = 0.02, pQ2 = 0.02, and Q2 = 0.761 (Figure S2). Therefore, there are predictive metabolites for classifying samples into one of the two groups. The VIP analysis allowed us to identify the metabolites that could explain the statistical difference between the two groups. This analysis found 102 metabolites with VIPs > 1. Metabolic pathway analysis of these 102 metabolites using MetaboAnalyst V5.0 (www.metaboanalyst.ca, accessed on 22 January 2023) and the KEGG database identified that phenylalanine metabolism had a significant impact.

#### 3.4.3. Plasma Comparison by Univariate Analysis

A student’s *t*-test revealed 108 metabolites with significantly different expression before or after sonoporation. After adjustment for multiple comparisons by the FDR test, 62 metabolites were retained. A volcano plot is shown in Supplementary Figure S3 to illustrate the dysregulation of the expression of these metabolites. 25 metabolites were dysregulated, of which 20 were up-regulated and 5 down-regulated. All the up- and down-regulated metabolites are presented in Supplementary Table S2. Using MetaboAnalyst 5.0, with the 108 significantly modified metabolites, two significantly impacted metabolism pathways were found: (1) D-glutamine and D-glutamate, and (2) arginine biosynthesis.

## 4. Discussion

In the present study, we validated the safety of sonoporation of the inner ear in a sheep model. No ototoxicity of sonoporation was observed on ABR thresholds, consistent with previous sonoporation studies of the inner ear in guinea pigs [26,28,29,30,31,32]. For sheep #3, the threshold for wave IV detection increased from 20 to 30 dB after sonoporation, but this value remained within normal-hearing levels. This increase may have been due to sonoporation, surgery, or measurement error. The ABR curves were sometimes difficult to interpret despite the use of subcutaneous detection electrodes. Our ABR recordings only assessed auditory function at high frequencies. Another approach to objectively assess would be distortion-product otoacoustic emissions (DPOAEs), which is a multi-frequency method. However, this method may not work because of the morphology of the sheep’s tortuous external ear canal, the milling-induced damage during mastoidectomy, and the presence of fluid (MBs, blood) in the middle ear, which may cause conductive hearing loss.

The ultrasound parameters used were chosen given their effectiveness in delivering therapeutic molecules in various contexts. For example, these parameters have been used to increase the permeability of the blood-brain barrier [45] or to increase the extravasation of local drug delivery to tumor tissue [48], as well as in previous studies regarding sonoporation of the RWM [26,27,28,29,30,31,32]. Some previous studies demonstrated increased steroid delivery to the inner ear using 1-MHz ultrasound waves [28] and increased safety (according to histology) with 3 rather than 5 one-minute consecutive exposures for the RWM [30]. In a previous study [49], these parameters induced inertial cavitation of Vevo MicroMarker^®^ MBs to stimulate adjacent cells and thus increase the delivery of therapeutic drugs. Note that, after activation by hand stirring, no further mixing method was used during sonoporation, as access to the middle ear was blocked by the transducer and head shaking risked MBs leakage. This could lead to the activation of only a fraction of the MBs by the ultrasound beam. In addition, the Vevo MicroMarker^®^ MBs used in our study are an ultrasound contrast agent indicated for preclinical ultrasound imaging. In previous studies, we reported that these MBs are more efficient than SonoVue^®^ MBs for drug and gene delivery in vitro using sonoporation [49,50]. If they are effective for enhanced delivery of drugs to the inner ear, and human use is intended, more studies will be necessary for regulatory approval.

Consistent with previous studies [29,30], sonoporation of the inner ear did not significantly increase the temperature in the middle ear. It is possible that the temperature could have increased because of interaction between the ultrasound waves and the bone structures of the middle ear, but this was not observed. The increase in temperature must not exceed the permissible toxicity threshold for ultrasound (1.5 °C [33]). To limit this effect, the sonoporation was applied for 1 min and repeated three times with 1 min between applications rather than 3 min of continuous stimulation.

This is the first study to investigate the safety of sonoporation of the inner ear by analysing the metabolome of perilymph from control and sonoporated ears. Several metabolites and metabolic pathways have been identified in the perilymph after inner ear trauma. Trinh et al. [43] reported that N-acetyl neuraminate increased in the metabolomic profiles of perilymph samples from cochlear implant patients with long durations of hearing loss, indicating the destruction of cell membranes. This hearing loss also induced a significant increase in the concentrations of several other metabolites, including glutaric acid, cystine, 2-propanoate, butanoate, and xanthine. In addition, Fujita et al. [51] investigated the metabolic consequences of auditory trauma in the perilymph of guinea pigs using metabolomics. They identified 12 metabolites whose concentrations were significantly altered: 3-hydroxy-butyrate, glycerol, fumaric acid, phosphate, and pyruvate + oxalacetic acid were significantly increased, while citric + isocitric acid, mannose, meso-erythritol, galactosamine, and inositol were significantly decreased. In a mouse model of auditory trauma, Ji et al. [52] reported that the glutamate, aspartate, purine, and alanine pathways were up-regulated in the perilymph, while the phenylalanine, tyrosine, and tryptophan pathways were down-regulated. In the present study, none of these metabolites or pathways were significantly changed in the perilymph of the sonoporated ear, suggesting that sonoporation did not cause damage to the inner ear under our experimental conditions. Theoretically, sonoporation could cause damage to RWM cells, especially when inertial cavitation of the MBs is reached, as it can cause intercellular ruptures, membranous pores, and cell destruction. 

Additional adverse events, such as facial palsy, could be induced by ultrasound application in the middle ear. We could not evaluate this possibility here, as sacrifice of the facial nerve was surgically necessary to gain access to the RWM for perilymph sampling. A long-term study of auditory responses and metabolomic perilymph profiles is needed to assess the complete innocuity of this technique. Our perilymph samples were collected approximately 30 min after the sonoporation procedure, which allowed for only short-term metabolomic effects.

Complementary to the metabolomic analysis of the perilymph, a metabolomic study of the plasma was undertaken by taking blood samples before and after sonoporation. This was done to further investigate metabolomic changes if differences were observed in the perilymph. The metabolomic analysis of serum after sonoporation revealed significant changes in the metabolic pathways of phenylalanine metabolism, D-glutamine and D-glutamate metabolism, and arginine biosynthesis. Phenylalanine is an essential amino acid that is converted to tyrosine and used in the biosynthesis of dopamine and noradrenaline. In our study, phenylalanine metabolism was activated, increasing these neuromodulators, which are notably secreted in response to exhaustion or stress. Glutamine is a non-essential amino acid, a substrate for the production of excitatory and inhibitory neurotransmitters (glutamate and GABA), but also a major source of energy for the nervous system. In our study, the D-glutamine and D-glutamate metabolism pathways were activated towards the production of 2-oxo-glutarate, which is used in the Krebs cycle for energy production. L-citrulline is the most dysregulated metabolite of the arginine pathway. It is also a precursor of the Krebs cycle and therefore contributes to energy production. The data used for metabolomic analysis of plasma should be interpreted with caution, as the blood sample taken before sonoporation was taken right after anesthetic induction. The anesthesia and surgical procedure may have contributed to the stress and energy consumption observed here. A second limitation is that a human KEGG database was used for the metabolomic analysis and pathway research. To our best knowledge, an ovine metabolomic database is not available.

Finally, the surgical technique used to access the inner ear here was a mastoidectomy, which was necessary to easily gain access to the round window, to deliver the ultrasound stimulation, and to collect perilymph. Other approaches to the round window could be developed with adapted ultrasound probes, as done for guinea pigs [29,31].

## 5. Conclusions

The present study established the safety of sonoporation of the inner ear in a sheep model. This procedure did not cause deafness, harmful heating of the inner ear, or metabolomic changes in the perilymph. Further experiments are required to investigate the efficacy of sonoporation for delivering therapeutic drugs to the inner ear in a sheep model.

## Figures and Tables

**Table 1:** ABR thresholds of wave IV detection are presented for each sheep before and after sonoporation session and for control side.

	Thresholds (dB)		
Sheep	Before Sonoporation	After Sonoporation	Control
#1	30	30	30
#2	30	30	30
#3	20	30	20
#4	20	20	20
#5	20	20	20
#6	20	20	20

**Table 2:** Latency measurements for Wave IV in milliseconds (ms) at 30 and 20 dB for each sheep before and after sonoporation, and for control side. # indicates missing values.

Sheep	Measurement	30 dB	20 dB
#1	Before sonoporation	4.49	#
	After sonoporation	5.22	#
	Control side	5.24	#
#2	Before sonoporation	4.9	#
	After sonoporation	4.75	#
	Control side	4.82	#
#3	Before sonoporation	4.74	5.10
	After sonoporation	4.75	#
	Control side	4.61	5.10
#4	Before sonoporation	4.36	4.59
	After sonoporation	4.35	4.85
	Control side	4.77	4.88
#5	Before sonoporation	4.69	5.15
	After sonoporation	5.10	5.40
	Control side	4.49	4.84
#6	Before sonoporation	4.83	5.08
	After sonoporation	4.93	5.50
	Control side	5.03	5.39

## Data Availability

The datasets generated during and/or analyzed during the current study are available in supplementary materials. Other data are available on reasonable request from the corresponding author.

## Supplementary Materials

The following supporting information can be downloaded at: https://www.mdpi.com/article/10.3390/pharmaceutics15020442/s1, Table S1. List of metabolites found in the perilymph on sonoporation and control sides. For each metabolite, the mean relative concentration and standard deviation are presented for the sonoporation side and control side; Table S2. List of down- and up-regulated metabolites in plasma after sonoporation with mean concentration and standard deviation before and after sonoporation, as well as *p*-values from Student’ *t*-test and after FDR adjustment and Fold-Change; Figure S1. Unsupervised multivariate analysis of perilymph comparing sonoporation and control groups. Score-plot of the PCA constructed from the metabolites found according to components p1 and p2. The control samples are represented in purple (C1 to C6) and the sonoporation samples are in green (S1 to S6). Each group is associated with an ellipse regrouping all its samples. The thicker dots correspond to the center of each ellipse; Figure S2. Supervised multivariate analysis of plasma comparing before and after sonoporation groups. PLS-DA constructed from the metabolites found according to components p1 and p2 with 100 permutations. The samples before sonoporation (Before) are represented in red (C1 to C6) and the samples after sonoporation (After) are represented in blue (D1 to D6). Each group is associated with an ellipse regrouping all its samples; Figure S3. Volcano-plot comparing the dysregulation of metabolites before and after sonoporation, with thresholds set at log2FC < −1 or >1 and adjusted *p*-value −10log > 1.3 (*p* < 0.05). The blue symbols represent down-regulated metabolites, the red symbols represent up-regulated metabolites, and the gray symbols represent no significant dysregulation.
