# Supplementary material for: Sonoporation of the Round Window Membrane on a Sheep Model: A Safety Study
Source: Pharmaceutics. 2023 Jan 29;15(2):442. doi: 10.3390/pharmaceutics15020442 (PMC9964975; doi:10.3390/pharmaceutics15020442)
Supplement: Supplementary file 1 [file pharmaceutics-15-00442-s001.zip › pharmaceutics-2090871-supplementary.pdf]

Supplementary Materials: Sonoporation of the Round Window Membrane on a Sheep Model: A Safety Study

Sandrine Kerneis, Jean-Michel Escoffre, John J. Galvin III, Ayache Bouakaz, Antoine Presset , Corentin Alix, Edward Oujagir, Antoine Lefèvre, Patrick Emond , Hélène Blasco and David Bakhos

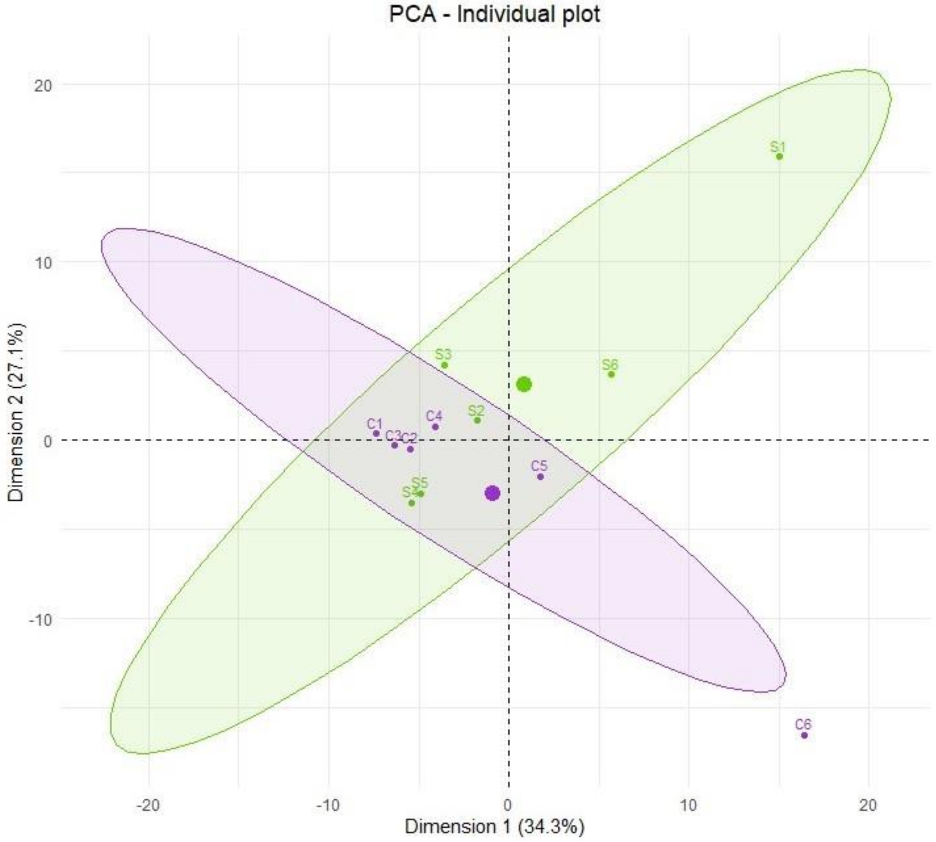

Figure S1. Unsupervised multivariate analysis of perilymph comparing sonoporation and control groups. Score-plot of the PCA constructed from the metabolites found according to components p1 and p2. The control samples are represented in purple (C1 to C6) and the sonoporation samples are in green (S1 to S6). Each group is associated with an ellipse regrouping all its samples. The thicker dots correspond to the center of each ellipse.

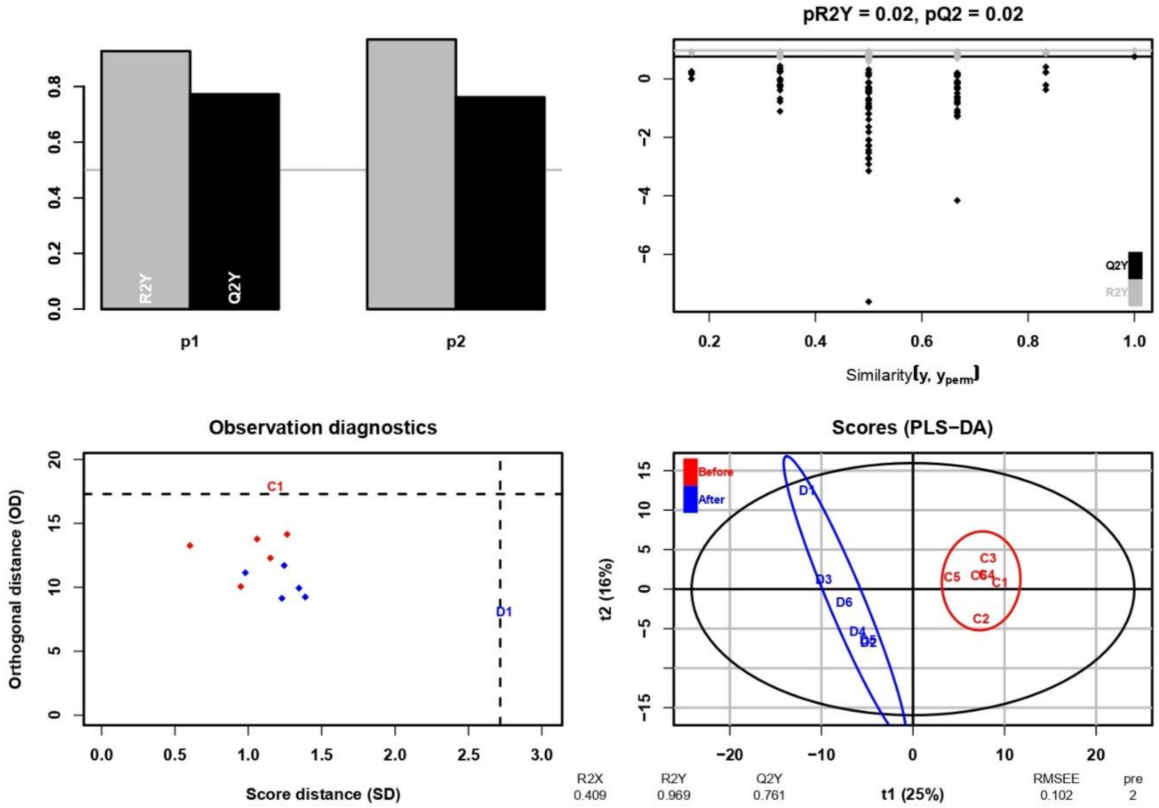

Figure S2. Supervised multivariate analysis of plasma comparing before and after sonoporation groups. PLS-DA constructed from the metabolites found according to components p1 and p2 with 100 permutations. The samples before sonoporation (Before) are represented in red (C1 to C6) and the samples after sonoporation (After) are represented in blue (D1 to D6). Each group is associated with an ellipse regrouping all its samples.

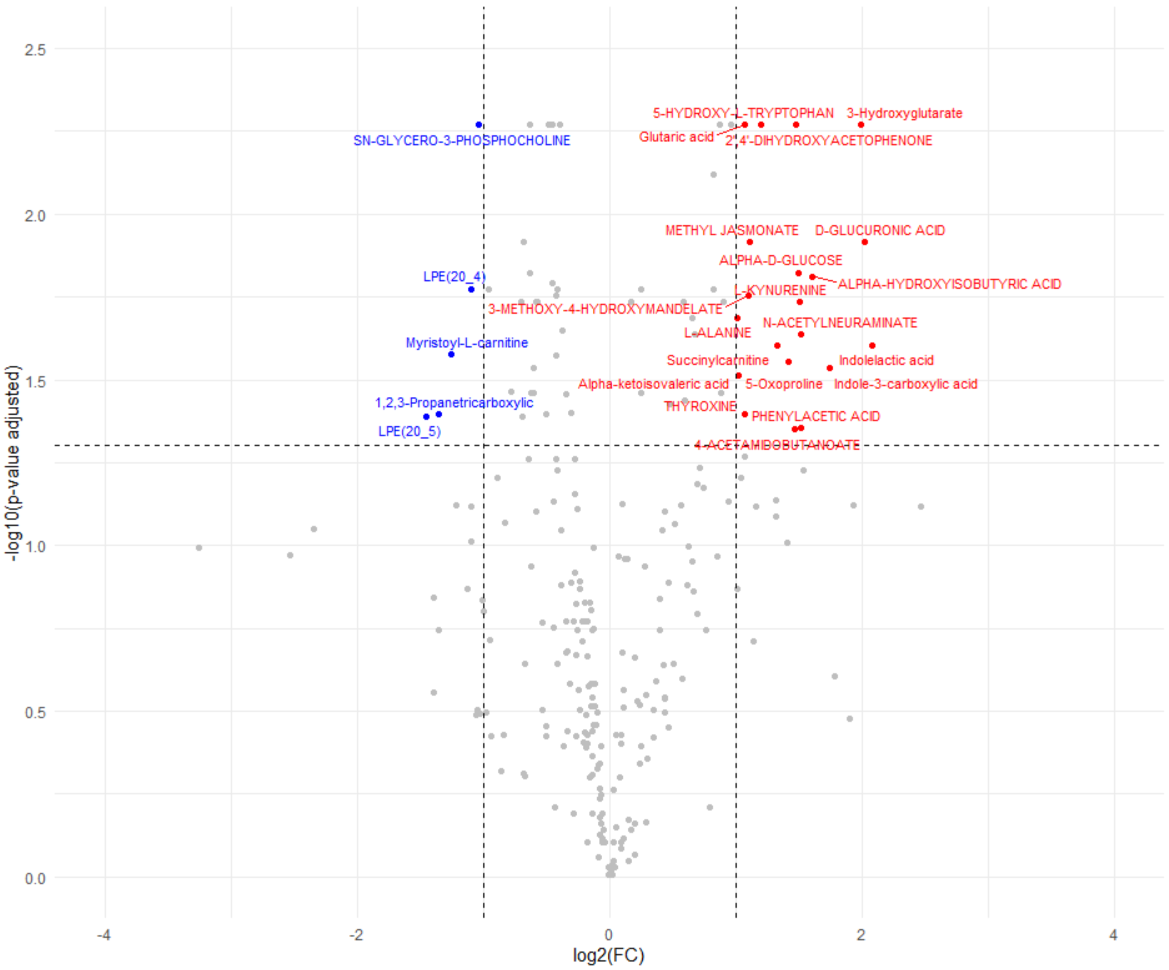

Figure S3. Volcano-plot comparing the dysregulation of metabolites before and after sonoporation, with thresholds set at log2FC < −1 or > 1 and adjusted *p*-value −10log >1.3 ($p < 0.05$). The blue symbols represent down-regulated metabolites, the red symbols represent up-regulated metabolites, and the gray symbols represent no significant dysregulation.

Table S1. List of metabolites found in the perilymph on sonoporation and control sides. For each metabolite, the mean relative concentration and standard deviation are presented for the sonoporation side and control side.

	Sonoporation ear		Control ear	
**Metabolites**	**Mean**	**Std.**	**Mean**	**Std.**
1,2,3-Propanetricarboxylic	0.009611	0.006626	0.011413	0.004348
12-Hydroxy-9-cis-octadecenoic acid	0.001434	0.001227	0.001270	0.000659
1-Aminocyclopropanecarboxylic acid	0.003072	0.001017	0.003343	0.000618
1-Methyladenosine	0.000103	0.000102	0.000243	0.000384
2',4'-Dihydroxyacetophenone	0.000292	0.000055	0.000387	0.000168
24-Hydroxycholesterol	0.000881	0.000377	0.001163	0.000408
3-(3-Methoxy-4hydroxyphenyl)lactate	0.000671	0.000311	0.000991	0.000379
3-hydroxyisobutyrate	0.007830	0.010110	0.005271	0.003982
3-Hydroxyoctanoic acid	0.001244	0.000166	0.001563	0.000697
3-Methyl-2-Oxovaleric acid	0.003150	0.002021	0.002732	0.000953
3-Methyladipic acid	0.000614	0.000109	0.000776	0.000257
3-Ureidopropionate	0.000054	0.000043	0.000075	0.000062
4-Guanidinobutanoate	0.000250	0.000176	0.000427	0.000388
4-Hydroxy-3-Methoxyphenylglycol	0.000904	0.000427	0.001250	0.000518

4-Hydroxy-L-Proline	0.001672	0.000191	0.001644	0.000380
4-Hydroxyphenyllactic Acid	0.000500	0.000400	0.000466	0.000169
4-Imidazoleacetic Acid	0.000213	0.000028	0.000211	0.000074
4-Methyl-2-Oxo-Pentanoic Acid	0.004315	0.002002	0.003665	0.001758
5-Methylcytosine	0.000480	0.000169	0.000684	0.000358
5-Oxoproline	0.012005	0.006194	0.011415	0.004887
7-Dehydrocholesterol	0.004184	0.002026	0.005900	0.002484
7-Ketodeoxycholic Acid	0.000125	0.000133	0.000070	0.000066
Adenosine	0.017501	0.028559	0.013550	0.011296
Allantoin	0.002745	0.002532	0.002379	0.000911
Alpha-Aminoadipate	0.000379	0.000436	0.000162	0.000069
Alpha-Aminobutyric Acid	0.000897	0.000700	0.000583	0.000352
Alpha-D-Glucose	0.194806	0.046757	0.204547	0.021442
Alpha-Hydroxyisobutyric Acid	0.006443	0.003729	0.005105	0.002349
Alpha-Ketoglutaric Acid	0.002477	0.004063	0.001466	0.002002
Azelaic Acid	0.014189	0.002090	0.018746	0.009367
Betaine	0.335625	0.095357	0.303323	0.055632
Bilirubin	0.000072	0.000077	0.000060	0.000079
Butanoylcarnitine	0.002620	0.002053	0.000589	0.000278
Cadaverine	0.000178	0.000078	0.000395	0.000193
Cholic Acid	0.000561	0.000615	0.000406	0.000392
Cinnamoylglycine-Trans	0.000407	0.000708	0.000238	0.000133
Citraconic Acid	0.001125	0.000382	0.001110	0.000449
Citramalate	0.003240	0.002419	0.002704	0.001507
Citrate	0.020535	0.010389	0.013660	0.005207
Citrulline	0.002543	0.002048	0.001808	0.000632
Creatine	0.147254	0.084118	0.131674	0.035294
Creatinine	0.271930	0.111264	0.355657	0.069099
Cytidine	0.001866	0.001764	0.001601	0.000697
Cytidine 5'-Diphosphate	0.000029	0.000014	0.000040	0.000020
Cytosine	0.003658	0.001599	0.002934	0.000481
Deoxycarnitine	0.001409	0.000735	0.000799	0.000328
Deoxycholic Acid	0.000282	0.000210	0.000192	0.000144
Deoxycytidine	0.000112	0.000045	0.000135	0.000069
Deoxyuridine	0.000184	0.000093	0.000193	0.000157
D-Glucosamine	0.006560	0.003101	0.007623	0.001068
D-Glucose 6-Phosphate	0.002325	0.003443	0.000756	0.000879
D-Glucuronic Acid	0.001503	0.001338	0.000981	0.000429
D-Lactose	0.001104	0.000906	0.001634	0.002701
Dodec-2-Enedioic Acid	0.000533	0.000145	0.000724	0.000341
D-Pantothenic Acid	0.000313	0.000188	0.000335	0.000131
D-Xylose	0.005288	0.001165	0.005466	0.000630
Epinephrine	0.011544	0.003696	0.011584	0.004620
Ethanolamine	0.000259	0.000154	0.000469	0.000241

Ethanolamine Phosphate	0.000236	0.000105	0.000261	0.000124
Fumaric Acid	0.000566	0.000856	0.000450	0.000675
Gluconic Acid	0.006915	0.005314	0.004640	0.001018
Glyceraldehyde 3-Phosphate Diethyl Acetal	0.000614	0.000344	0.000848	0.000300
Glycerate	0.002799	0.000704	0.002857	0.001082
Glycerol 2-Phosphate	0.004955	0.004894	0.003041	0.001202
Glycine	0.004727	0.000984	0.004762	0.001215
Glycocholic Acid	0.000347	0.000615	0.000157	0.000216
Glycodeoxycholic Acid	0.000187	0.000341	0.000049	0.000040
Glycolaldehyde Dimer	0.003106	0.000762	0.003207	0.000370
Glycolate	0.001601	0.000296	0.001717	0.000341
Guanine	0.005683	0.002300	0.004958	0.001501
Guanosine	0.000550	0.000303	0.000513	0.000270
Hexadecanoylcarnitine	0.001296	0.002585	0.000137	0.000084
Hexanoylcarnitine	0.000350	0.000382	0.000036	0.000017
Hippuric Acid	0.008340	0.010618	0.006179	0.003369
Histamine	0.000193	0.000112	0.000475	0.000263
Hydroxybutyrylcarnitine	0.002476	0.002301	0.000357	0.000238
Hypoxanthine	0.106630	0.033771	0.127808	0.049083
Indoxyl Sulfate	0.000448	0.000539	0.000246	0.000131
Inosine	0.004130	0.001644	0.004973	0.001975
Isocitric Acid	0.001431	0.000510	0.001608	0.000713
Lactate	0.497440	0.128904	0.531821	0.015342
L-Alanine	0.002338	0.002821	0.001295	0.000529
L-Anserine	0.000329	0.000324	0.000269	0.000115
L-Arginine	0.016953	0.011294	0.025355	0.007870
L-Asparagine	0.000476	0.000344	0.000280	0.000096
L-Aspartate	0.001120	0.000408	0.001621	0.002163
L-Carnitine	0.099809	0.061666	0.042855	0.016631
Leucine	0.000213	0.000181	0.000136	0.000050
L-Glutamic Acid	0.008455	0.006321	0.005808	0.003746
L-Glutamine	0.044834	0.006259	0.047316	0.012666
L-Histidine	0.004355	0.001779	0.004820	0.000802
L-Kynurenine	0.000656	0.000282	0.000730	0.000489
L-Lysine	0.003390	0.000779	0.003586	0.000464
L-Methionine	0.003040	0.000615	0.002992	0.000553
L-Ornithine	0.002856	0.002306	0.002044	0.000803
LPC(14_0)	0.000026	0.000022	0.000017	0.000019
LPC(16_0)	0.001771	0.001624	0.001518	0.001785
LPC(16_1)	0.000084	0.000075	0.000043	0.000056
LPC(18_0)	0.001446	0.001280	0.001229	0.001231
LPC(18_1)	0.001316	0.001119	0.000824	0.000897
LPC(18_2)	0.000467	0.000507	0.000269	0.000320
LPC(18_3)	0.000163	0.000176	0.000062	0.000085

LPC(20_4)	0.000161	0.000214	0.000127	0.000126
LPC(22_6)	0.000150	0.000184	0.000079	0.000103
L-Phenylalanine	0.001307	0.000744	0.001130	0.000357
L-Proline	0.022170	0.008178	0.017150	0.005232
L-Serine	0.002335	0.000675	0.002549	0.000694
L-Threonine	0.002316	0.000932	0.001975	0.000699
L-Tryptophan	0.001919	0.001546	0.001589	0.000760
L-Tyrosine	0.001450	0.000644	0.001204	0.000364
L-Valine	0.183791	0.036140	0.143511	0.031487
Malic Acid	0.002779	0.004733	0.001937	0.002818
Mannitol	0.009612	0.004220	0.012214	0.007072
Mesoxalate	0.000083	0.000053	0.000086	0.000035
Methyl Jasmonate	0.001600	0.000239	0.002019	0.000843
Myristoyl-L-Carnitine	0.000329	0.000655	0.000025	0.000020
N(Pai)-Methyl-L-Histidine	0.006046	0.001469	0.005756	0.000827
N6,N6,N6-Trimethyl-L-Lysine	0.012375	0.013274	0.012702	0.005437
N-Acetylglycine	0.001389	0.002289	0.000832	0.000646
N-Acetyl-L-Alanine	0.000366	0.000128	0.000444	0.000152
N-Acetyl-L-Aspartic Acid	0.000903	0.000375	0.006131	0.010005
N-Acetylneuraminate	0.000782	0.000383	0.001048	0.000347
N-Acetylputrescine	0.000154	0.000103	0.000311	0.000205
Nicotinamide	0.037819	0.016187	0.053950	0.011323
Nicotinate	0.001337	0.000802	0.002185	0.000450
Octadecanoylcarnitine	0.000921	0.001621	0.000207	0.000140
Octanoyl-L-Carnitine	0.000130	0.000191	0.000019	0.000005
Oleoylcarnitine	0.001413	0.002628	0.000306	0.000476
Omega-Hydroxydodecanoic Acid	0.000425	0.000164	0.000428	0.000199
Ophthalmic Acid	0.001351	0.002527	0.001006	0.000987
PC(30_0)	0.000028	0.000013	0.000035	0.000027
PC(31_0)	0.000038	0.000014	0.000039	0.000026
PC(32_0)	0.000110	0.000042	0.000173	0.000151
PC(32_1)	0.000110	0.000034	0.000136	0.000098
PC(32_2)	0.000014	0.000008	0.000026	0.000036
PC(33_0)	0.000038	0.000012	0.000044	0.000033
PC(33_1)	0.000120	0.000046	0.000117	0.000092
PC(33_2)	0.000059	0.000023	0.000051	0.000041
PC(34_1)	0.001545	0.000537	0.002143	0.002212
PC(34_2)	0.001458	0.000589	0.001463	0.001435
PC(34_3)	0.000545	0.000377	0.000431	0.000447
PC(35_1)	0.000161	0.000048	0.000186	0.000153
PC(35_2)	0.000194	0.000058	0.000199	0.000175
PC(35_3)	0.000088	0.000049	0.000075	0.000077
PC(36_1)	0.000713	0.000190	0.000987	0.000916
PC(36_2)	0.001261	0.000342	0.001519	0.001286

PC(36_3)	0.000842	0.000322	0.000938	0.000863
PC(36_4)	0.000423	0.000167	0.000489	0.000436
PC(36_5)	0.000281	0.000209	0.000271	0.000320
PC(36_6)	0.000028	0.000031	0.000017	0.000023
PC(37_4)	0.000065	0.000020	0.000069	0.000067
PC(37_5)	0.000057	0.000027	0.000059	0.000064
PC(38_2)	0.000038	0.000011	0.000047	0.000046
PC(38_3)	0.000145	0.000050	0.000144	0.000122
PC(38_5)	0.001008	0.000536	0.001116	0.001157
PC(38_6)	0.000465	0.000194	0.000574	0.000660
PC(38_7)	0.000041	0.000033	0.000032	0.000038
PC(39_5)	0.000060	0.000022	0.000065	0.000065
PC(39_6)	0.000051	0.000014	0.000060	0.000057
PC(40_4)	0.000045	0.000021	0.000057	0.000063
PC(40_5)	0.000315	0.000144	0.000384	0.000384
PC(40_6)	0.000370	0.000082	0.000524	0.000581
PC(40_7)	0.000082	0.000023	0.000104	0.000113
PC(40_8)	0.000054	0.000049	0.000044	0.000058
PC(40_9)	0.000022	0.000022	0.000019	0.000028
PE(18_1__18_1)	0.000013	0.000010	0.000044	0.000057
Phosphocholine	0.001697	0.000758	0.002737	0.002648
Propanoylcarnitine	0.004531	0.003786	0.001140	0.000417
Pyridoxamine	0.000757	0.000221	0.000802	0.000163
Quinoline	0.001027	0.000386	0.001127	0.000334
Riboflavin	0.000009	0.000006	0.000006	0.000002
Sarcosine	0.001521	0.000647	0.001444	0.000378
Sitosterol	0.001076	0.000488	0.001466	0.000472
SM(32_1)	0.000021	0.000005	0.000025	0.000024
SM(33_1)	0.000076	0.000017	0.000096	0.000089
SM(34_1)	0.000695	0.000115	0.001014	0.000835
SM(34_2)	0.000074	0.000025	0.000097	0.000098
SM(35_1)	0.000074	0.000012	0.000107	0.000090
SM(36_1)	0.000074	0.000035	0.000115	0.000053
SM(36_2)	0.000032	0.000004	0.000047	0.000037
SM(40_2)	0.000008	0.000002	0.000015	0.000014
SM(41_2)	0.000020	0.000007	0.000042	0.000040
SM(42_2)	0.000097	0.000063	0.000290	0.000286
SM(42_3)	0.000020	0.000013	0.000052	0.000050
Sn-Glycero-3-Phosphocholine	0.000808	0.000429	0.001168	0.001044
Suberic Acid	0.004365	0.000606	0.006085	0.003124
Succinic Acid	0.005313	0.007442	0.009207	0.018522
Taurine	0.049184	0.060318	0.016710	0.010733
Taurochenodesoxycholic Acid	0.000249	0.000234	0.000089	0.000069
Taurocholic Acid	0.000588	0.000622	0.000455	0.000867

Tetradecenoyl-L-Carnitine	0.000156	0.000287	0.000021	0.000016
Thymidine	0.000412	0.000205	0.000260	0.000220
Trans-Cinnamate	0.011609	0.005342	0.016196	0.005898
Uracil	0.000985	0.000257	0.001257	0.000386
Uridine	0.002860	0.001863	0.002339	0.000846
Urocanic Acid	0.000475	0.000179	0.000534	0.000255
Valeryl-L-Carnitine	0.000910	0.000583	0.000395	0.000050
Xanthine	0.000721	0.000872	0.000718	0.000625

Table S2. List of down- and up-regulated metabolites in plasma after sonoporation with mean concentration and standard deviation before and after sonoporation, as well as p-values from Student's t-test and after FDR adjustment and Fold-Change.

Metabolite	Before sonoporation		After sonoporation		p-value	adj p-value	Fold-Change	Expression
	Mean	Std.	Mean	Std.				
1,2,3-Propanetricarboxylic	0.004488	0.002029	0.001753	0.000670	0.008721	0.040147	0.390705	Down
2',4'-Dihydroxyacetophenone	0.000055	0.000022	0.000152	0.000026	0.000210	0.005384	2.787366	Up
3-Hydroxyglutarate	0.000926	0.000197	0.003684	0.000623	0.000214	0.005384	3.980310	Up
3-Methoxy-4-Hydroxymandelate	0.000059	0.000013	0.000126	0.000026	0.001653	0.017577	2.139103	Up
4-Acetamidobutanoate	0.000203	0.000054	0.000563	0.000250	0.010355	0.044594	2.772105	Up
5-Hydroxy-L-Tryptophan	0.000098	0.000054	0.000225	0.000060	0.000060	0.005384	2.299254	Up
5-Oxoproline	0.021581	0.010416	0.057686	0.026034	0.004498	0.027929	2.672940	Up
Alpha-D-Glucose	0.004168	0.000967	0.011795	0.002820	0.000923	0.015007	2.830014	Up
Alpha-Hydroxyisobutyric Acid	0.010324	0.005559	0.031441	0.012177	0.001040	0.015434	3.045515	Up
Alpha-Ketoisovaleric Acid	0.003306	0.001685	0.006699	0.001484	0.005277	0.030627	2.026284	Up
D-Glucuronic Acid	0.000521	0.000371	0.002115	0.000300	0.000610	0.012178	4.056619	Up
Glutaric Acid	0.000295	0.000155	0.000619	0.000119	0.000082	0.005384	2.095788	Up
Indole-3-Carboxylic Acid	0.000016	0.000004	0.000052	0.000016	0.004924	0.029216	3.358259	Up
Indolelactic Acid	0.000407	0.000126	0.001718	0.000526	0.003641	0.024924	4.219984	Up
L-Alanine	0.000513	0.000110	0.001031	0.000281	0.002693	0.020544	2.008565	Up
L-Kynurenine	0.000226	0.000136	0.000643	0.000278	0.002273	0.018388	2.842316	Up
LPE(20_4)	0.000002	0.000000	0.000001	0.000000	0.001509	0.016790	0.467395	Down
LPE(20_5)	0.000006	0.000002	0.000002	0.000001	0.009151	0.040720	0.364567	Down
Methyl Jasmonate	0.000370	0.000044	0.000801	0.000124	0.000670	0.012178	2.162471	Up
Myristoyl-L-Carnitine	0.000100	0.000053	0.000042	0.000028	0.004082	0.026580	0.417885	Down
N-Acetylneuraminate	0.000111	0.000031	0.000316	0.000093	0.003286	0.023089	2.859323	Up
Phenylacetic Acid	0.000039	0.000009	0.000111	0.000044	0.010114	0.044268	2.855621	Up
Sn-Glycero-3-Phosphocholine	0.001641	0.000243	0.000801	0.000165	0.000024	0.005384	0.487717	Down
Succinylcarnitine	0.000006	0.000004	0.000015	0.000002	0.003741	0.024974	2.515806	Up
Thyroxine	0.000103	0.000025	0.000217	0.000087	0.008574	0.040147	2.104058	Up

Legend. Std: Standard deviation; LPE: Lysophosphatidylethanolamine.
